# Effect of a Fine Fraction on Dynamic Properties of Recycled Concrete Aggregate as a Special Anthropogenic Soil

**DOI:** 10.3390/ma16144986

**Published:** 2023-07-13

**Authors:** Katarzyna Gabryś, Raimondas Šadzevičius, Midona Dapkienė, Dainius Ramukevičius, Wojciech Sas

**Affiliations:** 1 SGGW Water Centre, Warsaw University of Life Sciences—SGGW, 02787 Warsaw, Poland; wojciech_sas@sggw.edu.pl; 2 Institute of Hydraulic Engineering, Vytautas Magnus University Agriculture Academy, 53361 Kaunas, Lithuania; midona.dapkiene@vdu.lt (M.D.); dainius.ramukevicius@vdu.lt (D.R.)

**Keywords:** recycled concrete aggregate, recycling, shear modulus, damping ratio, resonant column test

## Abstract

The literature confirms that fine recycled concrete aggregate (fRCA) can be used as a replacement for natural soil in new concrete, offering many advantages. Despite these advantages, there are also critical barriers to the development of fRCA in new mixes. Among these, the first challenge is the variability of fRCA properties, in both physical, chemical, and mechanical terms. Many individual studies have been carried out on different RCA or fRCA properties, but little investigative work has been performed to analyze their dynamic properties. Therefore, the influence of the non-cohesive fine fraction content of RCA on the dynamic properties of this waste material, when used as a specific anthropogenic soil, has been studied in laboratory conditions, employing a standard resonant column apparatus, as well as piezoelectric elements. In the present research, special emphasis has been placed on the dynamic shear modulus, dynamic damping ratio, small-strain shear modulus, and small-strain damping ratio, as well as shear modulus degradation G(γ)/G_max_, the damping ratio increase D(γ)/D_min_, and the threshold shear strain amplitudes γ_tl_ and γ_tv_. Artificially prepared fRCAs with varying fine fraction contents (0% ≤ FF ≤ 30%, within increments of 5%) have been tested at different pressures (p′ = 90, 180, and 270 kPa) and relative densities of D_r_ > 65%. This study also examined the effect of two tamping-based sample preparation methods, i.e., dry and wet tamping. The results presented herein indicate that the analyzed anthropogenic material, although derived from concrete and produced by human activities, behaves very similarly to natural aggregate when subjected to dynamic loading. The introduction of a fine fraction content to fRCA leads to changes in the dynamic properties of the tested mixture. Concrete material with lower stiffness but, at the same time, with stronger damping properties can be obtained. A fine fraction content of at least 30% is sufficient to cause a significant loss of stiffness and, at the same time, a significant increase in the damping properties of the mixture. This study can serve as a reference for designing fRCA mixtures in engineering applications.

## 1. Introduction

Aggregate is a basic raw material that has been used by mankind since almost the beginning of civilization and housing development. Demand for aggregate is constantly increasing, especially in the construction industry. The production and consumption of aggregate in Europe account for over 4 billion tons of material. In EU countries, the vast majority of aggregates come from natural deposits [1], which, even though they are quite common on Earth, are a deficit material. In this situation, it seems reasonable to use recycled materials, e.g., construction waste, to reduce the depletion of natural aggregate (NA) [2]. 

The International Union of Laboratories and Experts in Construction Materials, Systems, and Structures (RILEM) distinguishes three types of recycled aggregate [3,4]:

- Type I—material derived from masonry elements,
- Type II—material derived from concrete elements,
- Type III—material that is a mixture of at least 80% natural aggregate and a maximum of 20% recycled aggregate.

Recycled concrete aggregate (RCA) is divided into three classes, depending on its applicability:

- Class A—recycled aggregate for concrete, with a wide range of applications, including marine and environmental structures;
- Class B—this covers the majority of recycled aggregates, used in combination with natural aggregate, and is suitable for concrete with moderate conditions of exposure;
- Class C—recycled aggregate, which is suitable for concretes with only the mildest level of exposure [5].

Several seminal works on the utilization of recycled aggregate in concrete were published before 1996. Buck, in his work from 1977 [6], reports the beginning of RCA exploitation until the end of the Second World War, when there was excessive demolition of buildings and roads, and a great need to dispose of the resulting waste and rebuild Europe. After this sudden need to recycle concrete, the use of RCA declined. Similar references can be found in the work of Nixon [7]. In the 1970s, the United States began to reuse RCA in non-structural applications, such as foundations, fill, and base material [8]. From 1996 to 2002, the main body of research on the use of recycled concrete aggregate (RCA) was developed, as these materials reflected the highest and most consistent quality of all the recycled aggregates available at that time. As sustainable building practices have become more widespread, the use of RCA concrete has been the subject of a considerable amount of research over the last few decades [9]. However, over the past few years, it has become apparent that the perception of and trends toward the use of recycled aggregate in concrete are growing worldwide, thereby reducing the environmental impact of landfill and virgin aggregate extraction and making construction projects more economical and environmentally sustainable [10]. Some of the main negative environmental issues associated with construction are summarized in the Oikonomou study [11]; the large-scale application of RCA can help to reuse waste materials and prevent more NA from being harvested. 

Recycled concrete aggregate (RCA), i.e., concrete crushed into appropriate fractions from existing concrete and reinforced concrete structures, concrete pavements, road and airport surfaces, curbs, and concrete blocks, is a recycled material that is used more and more often as an alternative in civil engineering activities [12]. The current prevalence of RCA and the current scale of demolition and refurbishment in the construction industry indicate that this will continue to be a potential source of material for the construction of earth structures for many years to come. However, RCA still requires research and confirmation of its physical and mechanical properties in a complex range of loading conditions, especially in the range of dynamic and cyclic loads. 

The increasingly common use of RCA for road foundations, working platforms, or soft soil improvement (dynamic replacement), among other applications, has forced researchers to determine its dynamic properties in terms of the range of small deformations [13]. The response of the soil under dynamic loading is strongly controlled by the mechanical properties of the soil, which include parameters such as the small-strain shear stiffness (G_max_), the shear modulus (G), and its degradation with increasing shear strain (G/G_max_ = f(γ)), the small-strain damping ratio (D_min_), the damping ratio (D), and its variation with the level of strain (D/D_min_ = f(γ)). Of these, the shear modulus and the damping effect are the most important material properties that can be used to characterize the dynamic behavior of soils [14]. 

The authors’ current research focuses on studying artificially prepared fine recycled concrete aggregate (fRCA) mixtures in the laboratory. Fine recycled concrete aggregate (fRCA) (with a particle size smaller than 4 mm) is produced by the repeated crushing of concrete rubble [15]. Currently, fRCA is used in low-grade applications such as the replacement of natural sand in cementitious renders and masonry mortars, for road construction, and as backfill for geosynthetic reinforced structures and soil stabilization [16]. There are some research reports concerning the use of fRCA as a sustainable substitute for natural sand and cement in structural concrete. These reports contain the results of tests made on laboratory crushed mortars and concrete. However, this is a material that differs from actual recycled concrete from the outset [16]. Furthermore, investigations in recent years have highlighted several issues with the use of fRCA in terms of the fresh and hardened properties of new concrete [17]. Debied et al. [18] observed that the high level of water absorption by fRCA can reduce the workability of the resulting concrete. The adhering mortar introduces additional fines to the new concrete, resulting in the insertion of more interfacial transition zones, which, in turn, negatively affects the transport and mechanical properties of the finished concrete. Since fRCA is easily contaminated by chlorides and sulfates, this can significantly reduce the durability of new concrete. 

Studies and practical experience regarding the appropriate treatment and use of fRCAs are rather limited and/or inconclusive. Moreover, the quality control limits for the physical and chemical properties of RCA are widely different [19]. Finally, there is a lack of quality assessment standards, which is the main reason why fRCAs are not yet used in new concrete manufacturing outside the laboratory [16]. 

A significant volume of research work has been performed by various researchers on the various aspects of fine content in granular materials, especially in the case of sands and sometimes gravels. Certain summaries of the shear strength properties of sandy soil with fines can be found in the work of Kolay [20], whereas Goudarzy et al. [21] present a summary of various studies on the dynamic behavior of sandy soil with fines. Studies on the effect of fine content in waste materials, such as RCA, on concrete’s mechanical properties and, in particular, its dynamic characteristics are rather limited. In practice, both natural and anthropogenic soil deposits contain matrices with coarse and fine fractions, giving them friction, cohesiveness, and other parameters. At lower fine-particle contents, dry coarse-grained soil may demonstrate the usual dense or loose skeletal structure. Voids may be filled with these fine particles without significantly affecting the skeletal structure. However, in cases where larger quantities of fine particles with a larger surface area are used, the contact area given by coarse soil particles may decrease, changing the entire soil mass matrix and thereby affecting its mechanical parameters. In the case of the concrete aggregate itself, too high a proportion of fine fractions in the aggregate mix leads to an unjustified increase in the demand for cement (worsening the economic conditions for concrete production) and for water. It also causes the deterioration of many of its properties [15]. The question can thus be raised as to whether the dynamic properties of crushed concrete mixed with fine fraction also deteriorate/change. 

In light of the above remarks, this study was designed to investigate the effect of using fine-graded (FF) materials (passing through a 63-μm sieve) in prepared mixes on the dynamic properties of pure fRCA. In this paper, the dynamic behavior of the mixtures is investigated for an FF content of up to 30% per unit of weight, using a resonant column apparatus. This study is preliminary and is part of an extensive program including experimental trials, being carried out at the Water Center of the Warsaw University of Life Sciences (WULS), Poland, and at the Institute of Hydraulic Engineering of the Vytautas Magnus University Agriculture Academy (VMU), Lithuania. The program aims to investigate and quantify the efficiency and ability to improve soil and the soil structure response to dynamic and cyclic loading using waste materials. The characteristics of aggregates—physical, chemical, and mechanical—have been the subject of research in recent years, both in WULS and VMU. The collaboration has led to joint research activities, the results of which have been presented in joint publications [22,23,24]. 

## 2. Materials and Methods

### 2.1. Experimental Material

The subject of the present study was recycled aggregate—crushed concrete with a size of 0/31.5 mm, created by crushing concrete curbs originating from a demolition site in Warsaw, Poland, and delivered to the geotechnical laboratory at WULS in their crushed form. Prior to the geotechnical examination, the strength class of the concrete together with its selected physical and chemical properties were estimated. These tests were outsourced to an external company. The strength class of the analyzed RCA was determined to be from C16/20 to C30/35, according to the standard PN-EN 206-1 [25]. As a part of the laboratory experiments on the dynamic properties of this material, the recycled aggregate was crushed further and then separated into the following fractions: <0.063 mm, 0.063–0.125 mm, 0.125–0.25 mm, 0.25–0.50 mm, 0.50–1.0 mm, and 1.0–2.0 mm (using a set of graded sieves) [22]. 

In addition to the standard sieve analysis, the authors performed tests on the recycled aggregate to ascertain their physical and geometric properties. The results of these tests are presented in Table 1. 

For the present experiments, a series of six fRCA mixtures were prepared, containing different amounts of fine fraction aggregates. Six soil gradation curves were designed with fractions of between 0.015 and 2 mm, based on the mass share. For each composition, two materials (pure fRCA and the calculated amount of FF for this fRCA) were carefully mixed by shaking in a closed container. Four mixes were prepared in air-dry conditions, along with two mixes with optimal moisture content (OMC). Described below are the symbols by which the mixtures are designated:

- **M1**, **M2**, **M3**, **M4**—a group of mixtures in air-dry conditions, with a grain size of 0/2 and an FF content of RCA of 0%, 10%, 20%, and 30%, respectively;
- **M5**, **M6**—a group of moisturized mixtures with a grain size of 0/2 and an FF content of RCA of 5% and 15%, respectively.

**Figure 1:**
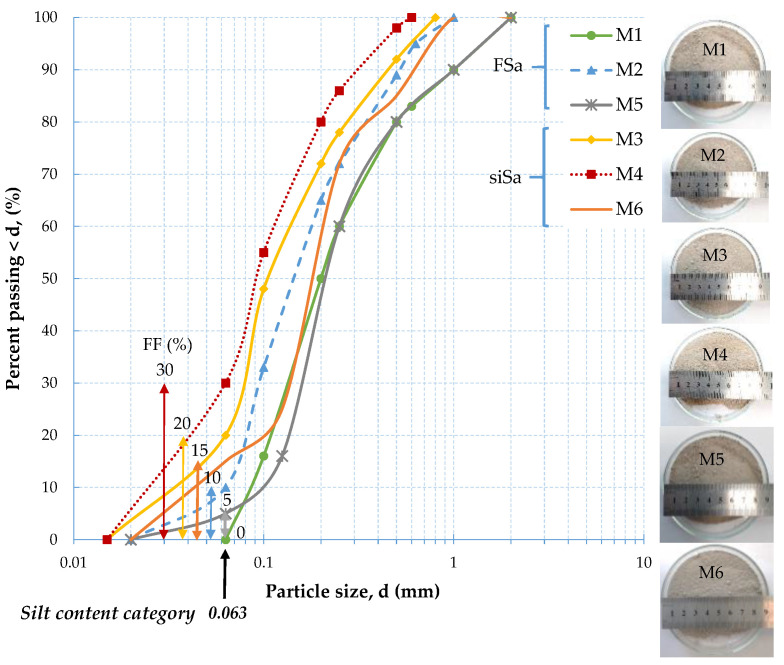
The tested materials’ particle size distribution curves, along with their photographs.

The particle size distribution curves of all six mixtures are shown in Figure 1, together with their photographs, and their main physical properties are provided in Table 2. When all the mixtures under test were evaluated for their grain size distribution coefficients (C_u_, C_c_, d_10_), the M1, M2, M5, and M6 blends could be classified as poorly graded fine SANDS (M1, M2, and M5 blends) (FF ≤ 10%) and SANDS with silt (M6 blend) (FF = 15%). Mixtures M3 and M4 were classified as well-graded SANDS with silt (FF > 15%). All the mixtures tested were of uniform particle size. 

**Figure 2:**
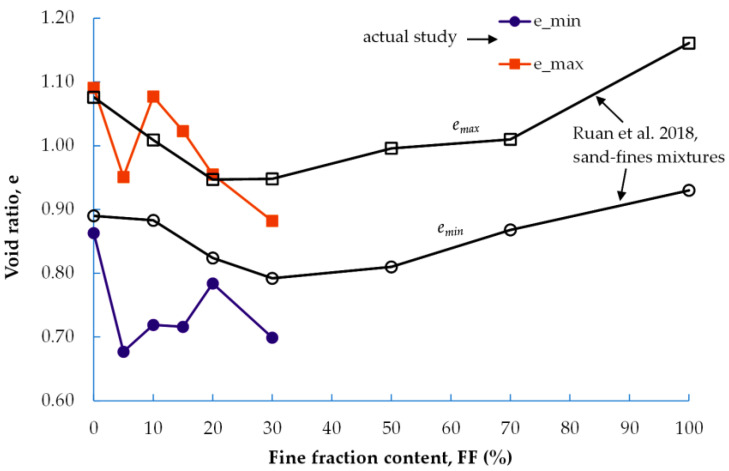
Maximum and minimum void ratios of the mixtures with different FF contents [37].

The e_max_ and e_min_ of the mixtures, determined through vibratory methods, specifically the metal vibrating forks method [36], are presented in Figure 2. This vibratory method enables the researcher to determine the maximum and minimum bulk density of the soil skeleton (ρ_d,max,_ and ρ_d,max_, respectively) of non-cohesive soils. Knowing the value of ρ_d,max_, the value of e_min_ can be calculated easily. Likewise, knowing the value of ρ_d,max_, the value of e_max_ can be established. As the fine fraction content of the anthropogenic soil being studied increases, the values of both e_max_ and e_min_ decrease (in the case of e_max_, the decrease is linear); however, this is only up to a certain point. This is a trend that can be observed in research on mixtures of natural soil, such as sand, which have a fine fraction. The current study was compared, among others, with research conducted by the team of Ruan et al. [37] on sand specimens mixed with fine content. As shown in Figure 2, for compositions with FF > 30% (approx.), there is an increase in the values of the void ratio. 

In this study, a series of specimens of around 70 mm in diameter and 140 mm high were prepared using dry and moist tamping methods. Four specimens (from M1 to M4) were prepared using the dry tamping method. The advantages of the dry tamping method have been reported by Zhu [38]. To shape another two specimens (M5 and M6), the moist tamping technique was adopted. Moist tamping has been widely employed in many experimental investigations regarding liquefaction [39]. As for the M5 and M6 compositions in this research, the technique consisted of mixing the fRCA particles with 5% and 15% of fRCA fine fraction, respectively, in a large blender in a partially submerged state, which was created by adding the appropriate amount of water (by weight) to the total dry mass. This appropriate amount of water was approximately equal to OMC. 

To achieve a uniform density, the under-compaction effect was induced by the energy transmitted by tamping the subsequent layers during specimen preparation [40]. Specimens were tamped in four equal layers at a free drop height of 10 to 50 mm. The number of blows was varied to prepare homogeneous samples. Special attention was paid to avoiding any particle segregation. Subsequently, the previously prepared mixture was placed in a split mold and then compacted carefully at a predetermined height to achieve the target density. Compaction was performed by hand, tamping with a graduated vertical rod and a small removable horizontal tab that controlled the desired height by abutting against the edges of the mold. 

The total mass that was required could be calculated readily, based on the knowledge of the potential volume of the specimen and fRCA specific gravity, maximum and minimum void ratios, and the desired relative density (*D_r_*). The authors aimed to make all specimens dense. Therefore, the relative density was consistently maintained at *D_r_* > 65%. 

The relative density of the specimens (*D_r_*) was determined by:

(1) $$D_{r}=\frac{\rho_{dmax}\cdot \left(\rho_{d}-\rho_{dmin}\right)}{\rho_{d}\cdot \left(\rho_{dmax}-\rho_{dmin}\right)}$$

where *ρ_d_*_,*max*_ and *ρ_d_*_,*min*_ are the maximum and minimum dry densities, respectively, of the specimen and *ρ_d_* is its controlled dry density. 

The maximum dry density and minimum dry density of the different mixtures were obtained via the vibratory method, using vibrating forks. For the M5 and M6 mixtures, *ρ_d_*_,*max*_ was found using the non-vibratory method (Proctor type). The soil mix was compacted with the use of normal Proctor’s compaction energy, equal to 0.59 J/cm^3^. The optimum moisture contents were set at 13.5% and 16% for the M5 and M6 mixes, respectively. 

### 2.2. Test Equipment and Test Procedure

**Figure 3:**
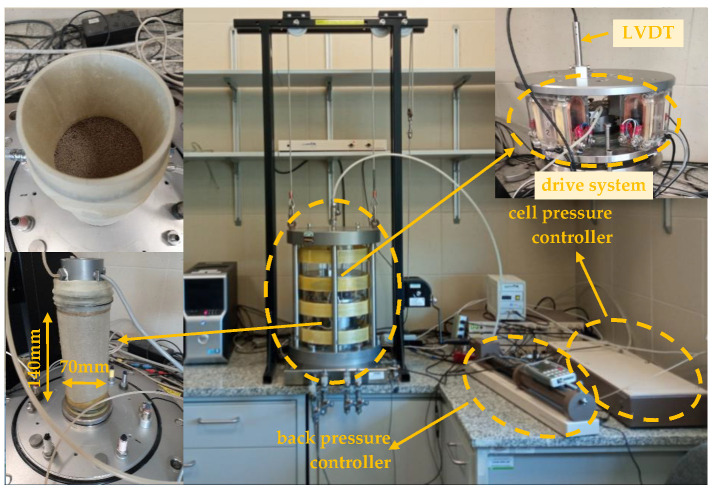
Typical sample diagram and experimental apparatus.

To characterize the dynamic properties of the mixtures, a GDS resonant column apparatus was used. Figure 3 shows photographs of the sample diagram and the experimental apparatus. Details of this instrumentation can be found, for example, in the manual of the GDS resonant column [41]. 

The resonant column test is based on the theory of wave propagation through prismatic bars. The subsequent analysis of the test data was described in detail by Drnevich [42] and used the ASTM D4015 standard [43].

Firstly, the sample was purged, i.e., a minimum confinement pressure was applied, and CO_2_ gas was passed through the sample for at least 30 min to expel any air bubbles from the equipment and facilitate the saturation process. The sample was then saturated with de-aired water and back pressure until the pore pressure coefficient (B-value) increased to a value greater than 0.95. Isotropic pressure consolidation was then carried out according to the target mean effective confining pressures (p′ = 90 kPa, 180 kPa, and 270 kPa) by controlling the radial stress applied to the specimen. It should be noted that consolidation was considered complete when the volume of back pressure in the specimen remained stable for 5 min after the drain valve was closed. Upon completion of the consolidation process, resonant column tests were carried out under undrained conditions [44]. 

Harmonic torsional excitation was applied to the top of the specimen by an electric motor in the electromagnetic drive system. A harmonic torsional load of constant amplitude was applied over a range of frequencies and the response curve (strain amplitude) was measured. The shear wave velocity (V_S_) was obtained by measuring the first mode resonant frequency. The small-strain shear modulus (G_max_) was calculated from this shear wave velocity and the soil density. The material damping (D) value was obtained from the free vibration decay after the forced vibration was stopped. After determining the G_max_ modulus and the D_min_ damping ratio, the cyclic torsional harmonic load amplitude was increased (from about 0.0006 V to 0.1 V) to obtain the strain-dependent shear modulus and damping values for a wide range of strains, from about 1 × 10^−4^% to about 3 × 10^−2^%. Each test was repeated 3 times.

In addition to the standard resonant column tests, in a comparative study to the G_max_ results, the piezoceramic bender elements (BEs) test was applied. The choice of the BE test was due to its having several advantages, such as ease of use, simplicity of principle, and non-destructive testing [45]. The G_max_ modulus was computed in the same way as in the case of the resonant column test. The difference, however, was in the estimation of V_S_. The V_S_ velocity was determined from the ratio of the shear wave propagation distance (L_tt_) to the propagation time (t). L_tt_ is the tip-to-tip distance between the transmitter and the receiver [45]. 

The piezoelectric bender elements were incorporated into the resonant column apparatus. Thus, the shear wave velocity measurements were conducted on the same specimens and under the same testing conditions, but with two different experimental techniques. Previous studies by the authors [46] indicated that the first time of arrival (FTA) technique is a reliable way to determine the propagation time accurately. Therefore, this method was selected to estimate V_S_ in the presented tests. The frequency range was chosen based on the literature review and the authors’ own experience. This article focuses primarily on shear wave velocity using one type of signal input: a sinusoidal signal. 

## 3. Experimental Results and Discussion

In this study, the resonant frequency (f) allowed the calculation of the shear wave velocity (V_S_), followed by the modulus G, as well as the coefficient D. The initial (maximum and small-strain) shear modulus (G_0_, G_max_) and the minimum damping ratio (D_min_) were found. In addition, the dependence of the *G* and *D* parameters on shear strain (γ) was plotted for each mixture using different values of p′. To compare how the curves G = f(γ) and D = f(γ) changed shape depending on the content of the fine fraction (FF) in the tested mixture, summarized figures were prepared. The stiffness and damping curves were normalized and the linear strain threshold (γ_tl_) was determined.

### 3.1. Shear Modulus and Damping Ratio as a Function of the Shear Strain of the Tested fRCA Mixtures

**Figure 4:**
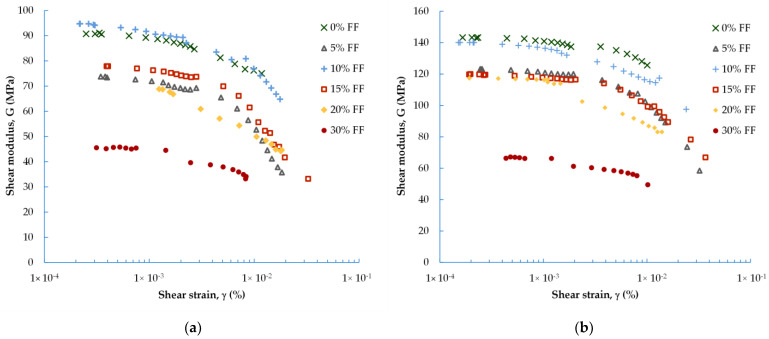

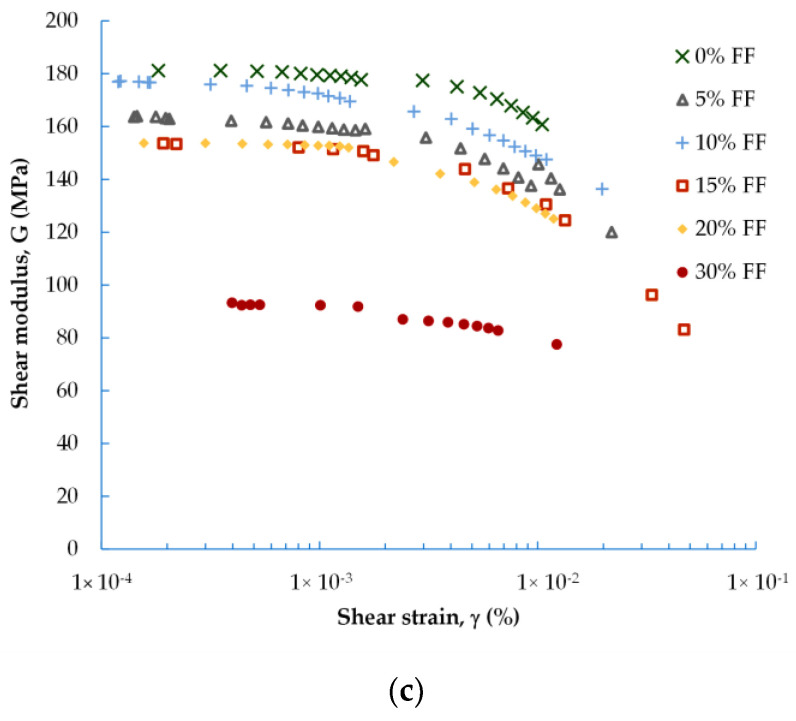
Effect of FF content on the dynamic shear modulus of fRCA mixtures under: (**a**) 90 kPa; (**b**) 180 kPa; (**c**) 270 kPa (filled symbols represent the dry tamping method, while empty symbols represent the wet tamping method).

**Figure 5:**
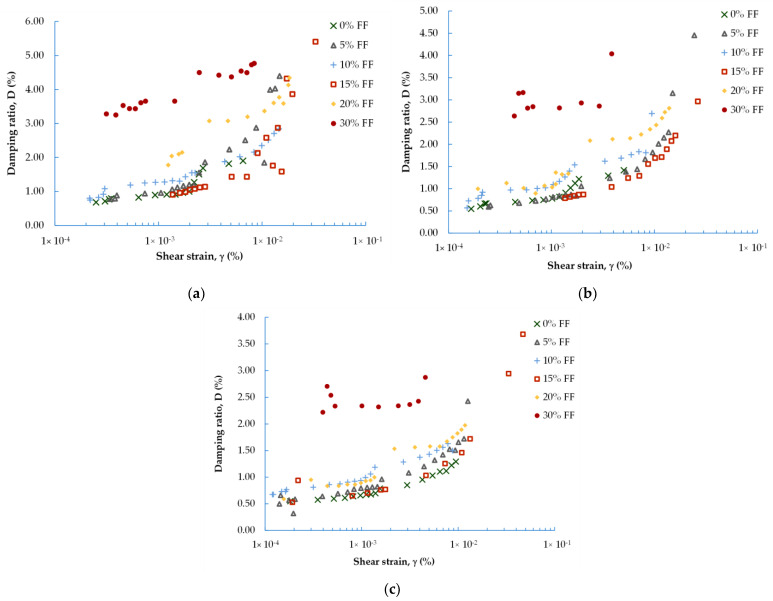
Effect of FF content on the dynamic damping ratio of fRCA mixtures under: (**a**) 90 kPa; (**b**) 180 kPa; (**c**) 270 kPa (filled symbols represent the dry tamping method, while empty symbols represent the wet tamping method).

In Figure 4 and Figure 5, the experimental data obtained for the dynamic shear modulus (Figure 4) and the dynamic damping ratio (Figure 5) under different evaluated conditions are compared. The well-known natural soil decrease in G and increase in D with increasing shear strain is noticeable here as well. The G = f(γ) and D = f(γ) curves follow regular courses and are similar to these for natural materials (such as natural aggregate). The maximum values, in the case of shear modulus, and minimum values, in the case of damping ratio, can be determined in the range of small strains (γ < 10^−3^%). In this research, it was possible to perform tests up to large strains at a level of around 0.05%. 

Regardless of the FF content, there is a strong relationship for each blend between the G and D parameters and the mean effective stress (p′). The increase in the stiffness of the mixture and the decrease in the mixture’s damping characteristic with increasing *p′* is comparable to the stiffness and damping characteristics of natural soils. It can be observed that the relationships for G = f(γ) and D = f(γ) could be well-fitted by a polynomial function. 

On average, for mixes with FF = 0%, the *G* values decrease by 14% with increasing γ, and, for mixes with FF > 0%, by about 26%. The smallest ΔG was recorded for the M4 composition, in air-dry samples with an FF > 0%. As shown in Figure 4, for those specimens prepared using the moist tamping method, the spread of results is greater. The dynamic shear modulus decreased on average by 46% with increasing strain (twice the decline compared to that of the air-dry specimens). Based on Figure 5, on average, for air-dry mixtures with FF < 30%, the D-values increased by about 65% as γ also increased. For the M4 mixture, with FF = 30%, the increase in damping ratio was the smallest, reaching approx. 30%. As discussed previously, in the case of moisturized mixtures, ΔD was equal to as high as 84% (20% more, compared to air-dry specimens). To sum up, with the additional 5% of FF content in the mixture, the G-modulus was reduced by about 5%, whereas the D-ratio was improved by about 22%.

Another factor that has a great influence on the stiffness and damping characteristics of natural aggregate, besides the strain level, is confining pressure as reported in the literature from the late 1960s, thanks to Hardin and Black [47]. The same factors determine the characteristics of the waste materials. For all the fRCA mixtures studied, it is clear that the greater the pressure, the greater the value of the G-modulus for the same strain level. The smaller the pressure, the greater the value of the D-ratio for the same strain level. 

As can be seen from Figure 4 and Figure 5, the results obtained for mixture M4, with FF = 30%, differ from the other mixtures. This may indicate that this percentage of fines content, i.e., FF ≥ 30%, is already sufficient to cause a significant loss of stiffness in the mix (between 26% for p′ = 90 kPa and 40% for p′ = 270 kPa), in comparison with blends characterized by a lower FF content. On the other hand, the addition of a percentage of fines of 30% also causes a significant increase in the damping properties of the mix (between 56% for p′ = 90 kPa and 73% for p′ = 270 kPa). Several studies reported the same observation [48], but this was for natural sandy soils. The studies explained that this variation in the mechanical properties with increasing FF content can be partly explained by the way in which the fines interact with the sand matrix (see the details in Carraro et al. [49]). If the fines are placed in the host soil matrix in such a way that they do not have well-developed contact with the host soil particles, shear waves, for example, will not be effectively transmitted through the fines, resulting in a lower G-modulus. 

Therefore, based on the literature [21], it appears that the skeleton structure of the mixture M4 is dominated by fines. The fines themselves and how they are arranged in the RCA matrix play a more important role than the RCA particles, even though this is still a mixture with a grain size of SAND wilt silt. Thus, the stiffness and damping of this specimen would be created by the fines.

**Figure 6:**
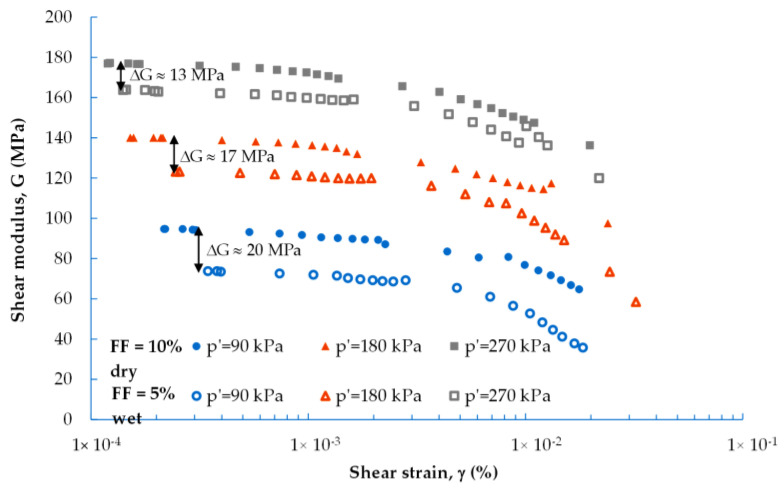
Effect of FF content on the dynamic shear modulus of the M2 (10% FF) and M5 (5% FF) mixtures under various mean effective stress scenarios.

**Figure 7:**
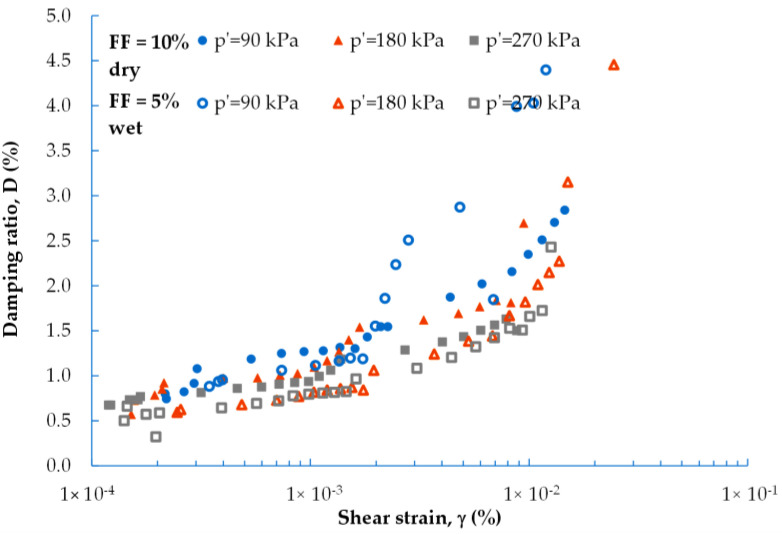
Effect of FF content on the dynamic damping ratio of the M2 (10% FF) and M5 (5% FF) mixtures under various mean effective stress scenarios.

The example comparison of the G(γ) and D(γ) data for two compositions prepared using dry (M2) and moist (M5) tamping techniques is shown in Figure 6 and Figure 7. The specimen prepared by dry tamping shows a higher dynamic shear modulus than the one prepared by moist tamping, although the content of FF in the dry mix is 5% higher than that in the wet mix. As illustrated in the previous figures, the reduction in soil stiffness increased with the FF content. In the opposite situation (Figure 6), there is a certain influence of moisture on the G-modulus of recycled concrete aggregate. The dynamic shear modulus of the M2 mixture is from 20 MPa (p′ = 90 kPa) to 13 MPa (p′ = 270 kPa), which is higher in comparison with the M5 mix. The addition of water during specimen preparation reduced the stiffness of the mixes, indicating their higher susceptibility to collapse, despite the lower contents of the fines. 

Regarding the dynamic damping ratio (Figure 7), at a shear strain amplitude of less than 1 × 10^−3^%, there is no distinguishable difference between the D-ratio values, regardless of mixture preparation and fine fraction content. However, with increasing γ, the level of medium strains and the sampling technique has some influence on the results obtained, especially for p′ = 90 kPa. Hence, at lower pressure, greater damping (by 1% ÷ 1.8%, on average) is characterized by moisturized compositions with a lower FF content. For higher pressures (p′ > 90 kPa), the increase in the D parameter according to the sampling technique is considerably less pronounced. Mojtahedzadeh et al. [50] reported that the dry-tamping method resulted in a higher damping ratio, which is also confirmed in this study of waste materials. 

### 3.2. Small-Strain Shear Modulus and Damping Ratio

**Figure 8:**
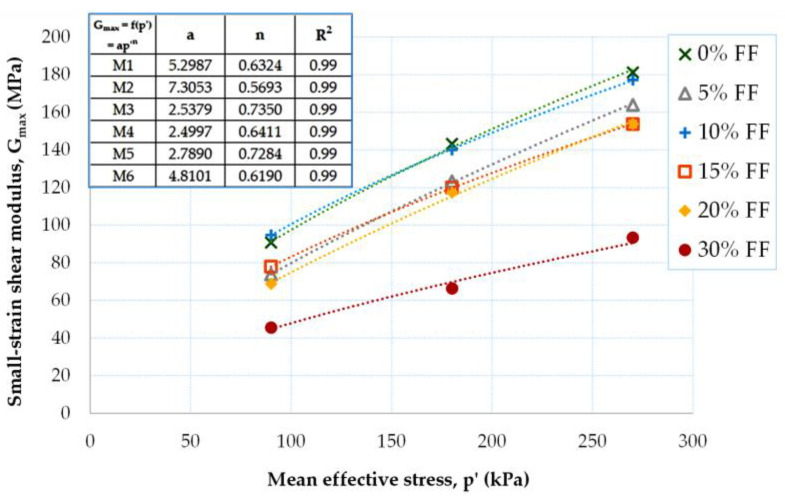
Effect of mean effective stress on the small-strain shear modulus of fRCA mixtures with various fine fraction contents.

Figure 8 shows the variation in the small-strain shear modulus as a function of p′. The dependence between G_max_ and p′ for all fRCA mixtures confirmed the power–law relationship (G_max_~p′^n^), which is typical for natural soils [51]. If the specimens have similar D_r_ values, the G_max_ of the mixtures with different FF contents increases with the increase of p′ at a uniform rate. This is comparable with the results for the silty sand mixtures tested by Salgado et al. [52]. The largest increase in G_max_ was recorded for the M6 mix (FF = 15%), by an average of 49%, and the M5 mix (FF = 5%), by an average of 44%. The G_max_ (p′) curves for M5 and M6 were characterized by the greatest slope. For the air-dry mixtures, ΔG_max_ was, on average, between 27% (M2) and 32% (M3). It is also worth mentioning that the moisturized blends have stiffness values that are quite similar to each other and are between the G_max_ values for FF = 10% and FF = 20%. 

Analysis of the data in Table 3 shows that the largest variation in G_max_ = f(p′) occurs when FF = 10% is exceeded. The small-strain shear modulus for mixtures M1 and M2 (homogeneous and even-grained material) shows very similar values. For an FF content > 10%, a decrease in the G_max_ values of approx. 85% is noted. The two values obtained in Table 3, with a negative sign for p′ = 90 kPa, deviate from the other results. They indicate that the mixture with FF = 10% has a higher G_max_ value than the pure RCA mixture, while the mixture with FF = 15% has a higher G_max_ value than the one with FF = 5%. In the remaining cases (and in most cases), an inverse relationship was observed, i.e., the lower the fines content, the higher the G_max_ parameter. 

**Figure 9:**
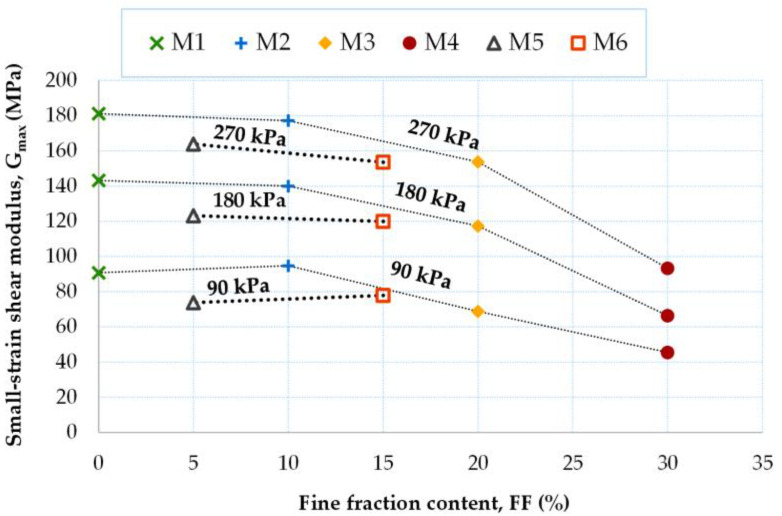
Small-strain shear modulus as a function of fine fraction content.

From the data in Figure 9, it is possible to quantify the reduction in G_max_ with the increasing FF content. For the air-dry mixtures, with an increase in the content of the fine fraction in the mixture, there is a reduction in the value of the G_max_-modulus, which, on average, is about 5%; the reduction in G_max_ with the increase in FF is greater for larger values of *p′* (e.g., for p′ = 270 kPa ΔG_max_ ≈ 30 MPa, for p′ = 180 kPa ΔG_max_ ≈ 26 MPa, for p′ = 90 kPa ΔG_max_ ≈ 15 MPa). For the moisturized mixtures, a reduction in G_max_ with increasing FF for lower pressures is not observed and is only seen for greater pressures, here equal to 270 kPa (a reduction of 10 MPa). Therefore, it appears that in the compositions made by dry tamping, the influence of FF on the stiffness of the anthropogenic soil mixtures is more pronounced. For further analysis and to draw relevant conclusions, more tests are needed. In Table 4, the variation in the small-strain shear modulus is presented with the variation in the mean effective stress for a given FF content. 

To summarize, when employing the moist tamping technique, the metastable structure of non-cohesive material with large voids is weakened [53]. Hence, less stiffness and greater susceptibility to failure of the material are seen (lower G_max_ values). Moreover, adding fine particles will further weaken this metastable structure. The decrease in small-strain stiffness with increasing fines content was explained by Wichtmann et al. [50], using the simulations of Radjai and Wolf [54] and Radjai et al. [55]. These simulations showed that strong and weak force chains are formed by the interparticle contacts in a polydisperse material, which demonstrates non-uniform packing of the granules, whereas force chains are more uniformly distributed in a monodisperse material, i.e., uniformly sized sand. Thus, in polydisperse packing, a large fraction of the grains participates only slightly in the transmission of external shear forces but reduces the void ratio by occupying space in the grain skeleton. Therefore, for a given void ratio, the overall shear stiffness of polydisperse packing is lower than that of monodisperse packing. A large number of fine particles (i.e., grain sizes < 0.063 mm) causes the packing to become more polydisperse, thus reducing its stiffness, as in the case of the fRCA mixtures studied here. In the present study, the limit of the fines content appears to be 30%, which has already been shown in Figure 4 and Figure 5. 

**Figure 10:**
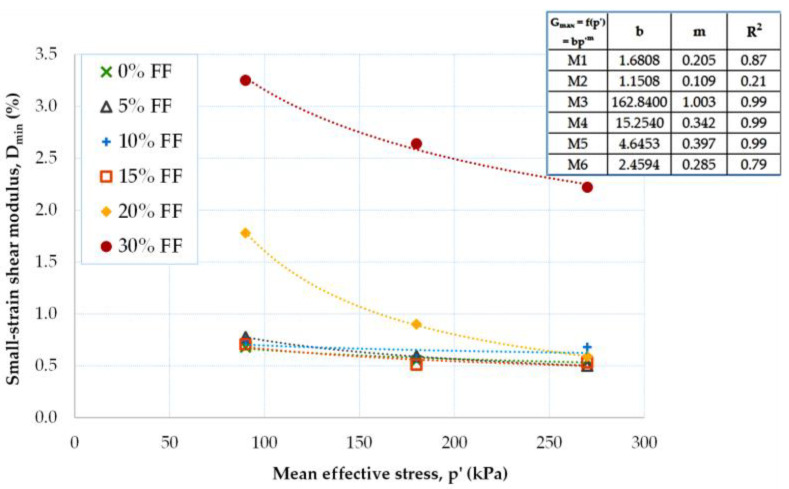
Effect of mean effective stress on the small-strain damping ratio of fRCA mixtures with various fine fraction contents.

The pressure dependence of the small-strain damping ratio for the fRCA mixtures being tested is depicted in Figure 10. The power–law relationship provides the best approximation of the experiments (D_min_ ~ p′^m^). However, the worst match was obtained for the M2 mix (R^2^ = 0.21). In the case of the fRCA mixtures reclaimed by dry tamping, the smallest decrease in *D_min_* with increasing pressure was observed for the M2 blend (FF = 10%) by an average of 2%, while the largest for the M3 blend (FD = 20%) was by an average of 42%. In the case of those mixtures created using the moist tamping method, the variation in the small-strain damping ratio was from 12%, for the specimen with a 15% FF content, to 20% for the specimen with a 5% content of FF. 

Analysis of the data in Table 5 shows that the biggest drop in the D_min_ = f(p′) curves occurred when the percentage of FF = 15% was exceeded. The D_min_ parameter for homogeneous, even-grained materials (mixes M1, M2, M5, and M6) exhibited very similar values. The difference in the dynamic damping ratio for those compositions where FF > 15% was registered at 94%.

**Figure 11:**
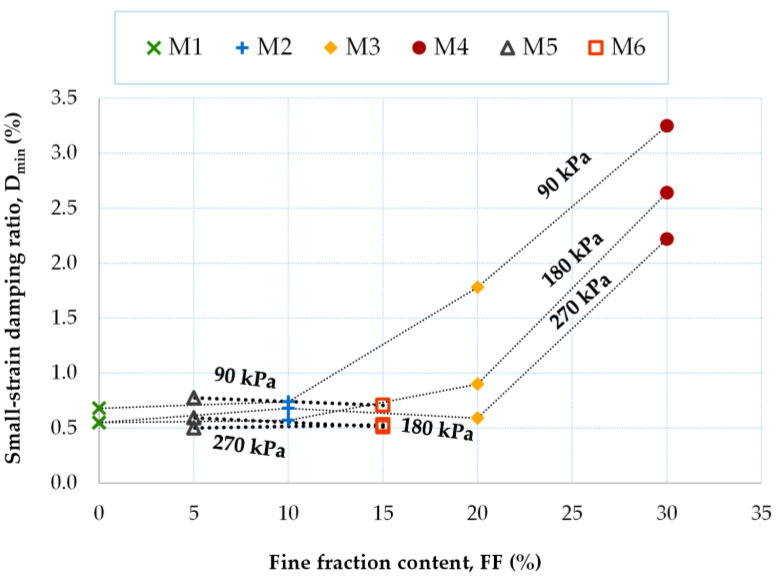
Small-strain damping ratio as a function of the fine fraction content.

The increase in the small-strain shear modulus with fine content can be seen in Figure 11. As the FF content in the air-dry mixtures increased, there was an equivalent increase in the value of D_min_, which was, on average, by about 22%. The increase in the D_min_ parameter with FF was greater for smaller values of *p′* (e.g., for p′ = 90 kPa, ΔD_min_ was less than 1%, and for p′ = 270 kPa, the ΔD_min_ value was about 0.5%). For the moisturized mixtures, lower dynamic damping ratios were measured for FF = 15% than for FF = 5%, which is opposite to the ratios for the air-dry mixtures. 

In Table 6, the variation in the small-strain damping ratio according to pressure for a given FF content can be traced. As noted earlier, poorly graded recycled concrete aggregates were characterized by similar damping ratio values, independently of the mean effective stress. For well-graded mixtures with higher FF content, ΔD_min_ was also higher. For these materials, the pressure dependence of the D_min_ parameter was somehow stronger for lower pressures. 

### 3.3. Normalized Shear Modulus and Damping Ratio

**Figure 12:**
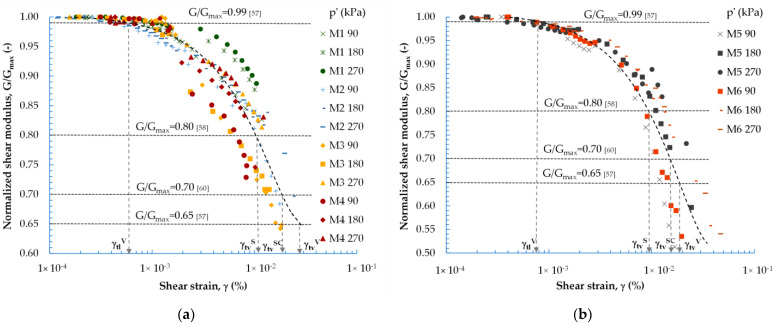
Normalized dynamic shear modulus vs. the shear strain for: (**a**) air-dry mixtures; (**b**) mixtures with OMC under various mean effective stress values, according to the fine fraction content (γ_tl_^V^—linear threshold shear strain [57]; γ_tv_^S^—volumetric cyclic threshold strain [58] γ_tv_^SC^—volumetric cyclic threshold strain [60]; γ_tv_^V^—volumetric cyclic threshold strain [57]).

**Figure 13:**
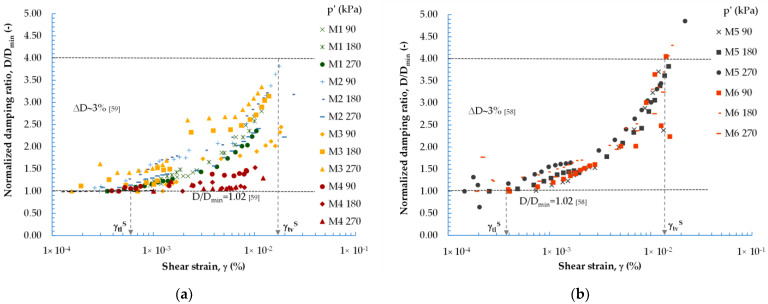
The normalized dynamic damping ratio vs. the shear strain for: (**a**) air-dry mixtures; (**b**) mixtures with OMC under various mean effective stress values, according to the fine fraction content (γ_tl_^S^—linear threshold strain [58].; γ_tv_^S^—volumetric cyclic threshold strain [58]).

For the fRCA mixtures tested, it is possible to present the normalized curves: the degradation of shear modulus curves (Figure 12) and the increase in damping ratio curves (Figure 13), with shearing strain amplitude for various values of p′. For any constant mean effective stress, an increase in FF content caused the values of G/G_max_ and D/D_min_ to decrease. This phenomenon was caused by the reduced stiffness of the material and indicates the more heterogeneous behavior of the mixture containing fine.

Using the criteria for natural soils, the strain thresholds [56] can also be determined for the studied mixtures of waste material. Thus, as with natural aggregate, for anthropogenic aggregate, it is also possible to identify the regimes of the linear elastic, non-linearly elastic, and plastic responses of the soil to dynamic loading. The linear threshold shear strain (γ_tl_) appears when the reduction in the G_max_ values is less than 1% and is represented by the strain level at a value of G/G_max_ = 0.99 (after Vucetic [57]) (Figure 12). In the case of damping (Figure 13), a strain level corresponding to γ_tl_ is equivalent to D/D_min_ = 1.02 (after Stokoe et al. [58]). For the tested air-dry mixtures, the linear threshold shear strain was 6 × 10^−4^% (Table 7). This agrees with the results from the study conducted by Jardine [59] for sands. Experiments performed on the moisturized specimens showed that this threshold shifted to the right for stiffness data and to the left for damping data. Nevertheless, γ_tl_ was in the range of values of less than 1 × 10^−3^%. 

The mixtures behaved non-linearly but still elastically once the linear threshold shear strain was exceeded. There are many theories about how to obtain the volumetric cyclic threshold strain (γ_tv_); hence, in this study, the conditions to evaluate γ_tv_ were chosen according to Stokoe et al. [58], Santos and Correia [60], and Vucetic [57]. Upon analyzing the results obtained, the volumetric cyclic threshold strain derived by Stokoe et al. [58] turned out to be the easiest to determine. According to Stokoe et al. [58], γ_tv_ occurs when the value of the shear modulus at its maximum value is about 80%, while the damping ratio increases by 3%. For the air-dry blends, the value of this threshold was estimated at an average of about 1.85 × 10^−2^%, whereas for the wet specimens, it was equal to 1.22 × 10^−2^%. 

**Figure 14:**
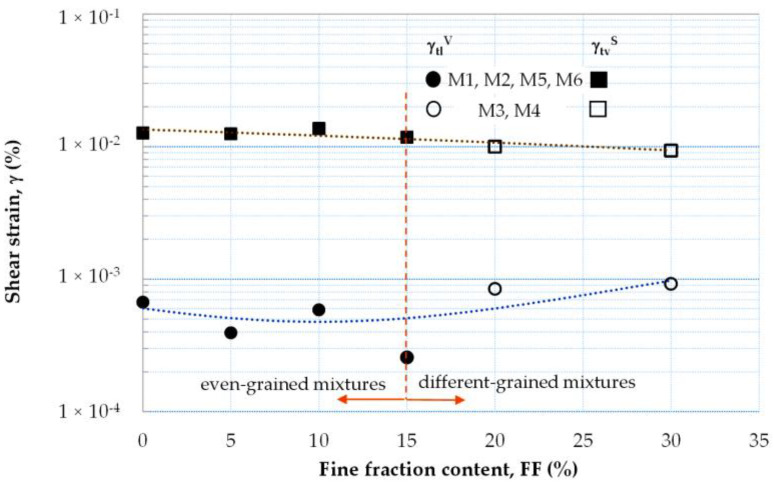
Threshold strain amplitudes, indicating either the transition from linearly elastic to the non-linearly elastic regime (γ_tl_^V^ = γ(G/G_max_ = 0.99)) or the beginning of plastic strains (γ_tl_^S^ = γ(G/G_max_ = 0.80)), depending on the fine fraction content.

The threshold shear strain amplitudes, which indicate the transition from linear to non-linear elastic behavior (γ_tl_^V^ = γ (G/G_max_ = 0.99)) or the onset of settlement (γ_tv_^S^), are plotted versus fine content in Figure 14. The γ_tl_^V^ and γ_tv_^S^ values that are given were the average of all the tests carried out on the fRCA mixtures. With the increase in FF content, the γ_tl_^V^ values in Figure 14 (dot symbols) increased, whereas the γ_tv_^S^ values (square symbols) decreased. For homogeneous, even-grained mixtures (FF ≤ 15%) (filled symbols), the volumetric cyclic thresholds demonstrated slightly higher values than those for heterogeneous, different-grained mixtures (FF > 15%) (open symbols). In the case of the linear thresholds, the situation was exactly the opposite. The mixtures with FF ≤ 15% exhibited lower γ_tl_^V^ values in comparison with those mixtures with greater fines contents. 

### 3.4. Comparison of G Using Resonant Column and Bender Elements Tests

**Figure 15:**
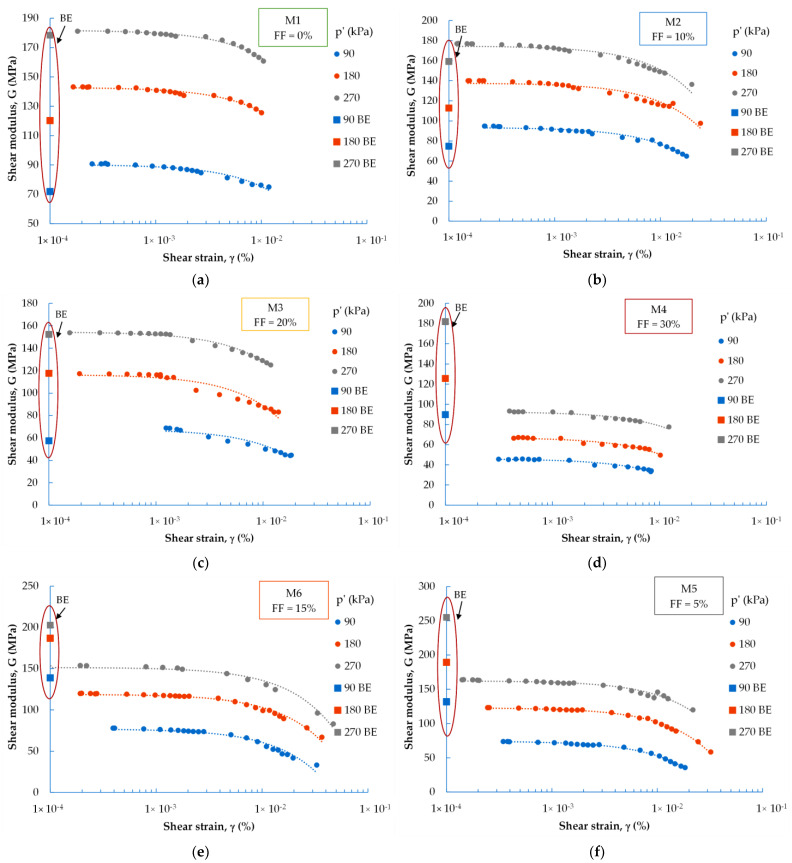
Dynamic shear modulus vs. the shear strain from the resonant column test and bender elements test: air-dry mixtures with (**a**) M1 mixture, (**b**) M2 mixture, (**c**) M3 mixture, (**d**) M4 mixture; mixtures with OMC, (**e**) M5 mixture and (**f**) M6 mixture.

The bender element (BE) technique has been broadly applied to determine the small-strain shear wave velocity of soil in practice, thanks to the simple, rapid performance of its application. To gain further insight into the dynamic properties of fine recycled concrete aggregate, after the resonant column (RC) tests, all the mixtures were measured with bender transducers. The test results are presented in Figure 15. 

In the case of the M1 and M2 mixtures (FF ≤ 10%), the G_max_ values measured by the resonant column test were higher by about 15% than their counterparts measured by the BE test. For the M3 mix (FF = 20%), similar values of G_max_ were obtained (after extending the G-curves from the resonant column test, the values of G_max_ measured by the BE test would fit perfectly). For the M4 blend (FF = 30%) and for the mixtures created by moist tamping, the G_max_ values measured by the BE test were higher; the difference in values is in favor of the BE test by about 48%. This may be because these two testing techniques cover a slightly different range of strains [61]; the results obtained from these tests in terms of strain values only overlap to a small extent. The small-strain shear modulus obtained from the resonant column test is usually determined at a relatively higher shear strain amplitude, whereas the BE test assumes a strain level of 1 × 10^−4^%. In addition, the coupling between the tested specimen and the top cap and bottom plate could be another factor causing a lower value for the G-parameter.

## 4. Conclusions

In this study, a series of isotropic, consolidated resonant column tests and bender elements tests were undertaken to investigate the dynamic behavior of fine recycled concrete aggregate as a waste material, and, on the other hand, as a special anthropogenic soil. The effects of various parameters, including the fine fraction content, shear strain amplitude range, mean effective stress, and two tamping-based mixture preparation methods were considered. The major outcomes of the study are as follows:

1. The fRCA, when tested under dynamic loading, behaved in a similar way to natural aggregate. The values of the dynamic shear modulus and dynamic damping ratio strongly depend on the level of shear strain and the average effective stress, regardless of the content of the fine fraction. The increase in the stiffness of the mixture and the decrease in the mixture damping with increasing p′ values were comparable to the stiffness and damping characteristics of natural soils.
2. A fines content of at least 30% had a significant effect on the stiffness and damping properties of the fRCA material tested.
3. The moist tamping method of sample preparation reduced the stiffness of mixtures, indicating their higher susceptibility to collapse, despite the lower contents of the fines. Considering the damping characteristic, at lower pressure, greater damping was considered for moisturized compositions, although this was with the inclusion of a lower FF content. For higher pressures, the increase in the D parameter according to the sampling technique was considerably less pronounced.
4. The air-dry even-grained mixtures (FF ≤ 10%) were characterized by similar G_max_ and D_min_ values. For mixtures with FF > 10%, there was a large decrease in the G_max_ values and an even greater increase in the D_min_ values. The reduction of the G_max_ modulus with FF was greater for the larger values of p′. The increase in the D_min_ ratio with FF was greater for the smaller values of p′. This was consistent with studies of natural soil mixtures.
5. For the moisturized mixtures, the reduction in G_max_ with increasing FF for lower pressures was not observed. Lower small-strain damping ratios were recorded for FF = 15% than for FF = 5%, which results were opposite to those of the air-dry mixtures. In the compositions made via dry tamping, the influence of FF on the stiffness of the anthropogenic soil mixtures was more pronounced.
6. When employing the moist tamping technique, the metastable structure of a non-cohesive material with large voids was weakened. Hence, we can establish the material with the lower G_max_ values; consequently, this was less stiff and more susceptible to failure. Moreover, with the addition of fine particles, this metastable structure was further weakened.
7. For the tested fRCA mixtures, it was possible to apply the criteria for strain regimes from the literature on a natural aggregate and determine the linear and volumetric cyclic threshold strains. The stiffness distribution of fRCA, similar to that of natural soil, exhibited non-linearity as a function of strain, most notably in the range of medium strains at (10^−3^ ÷ 10^−2^%). A slight increase in the *γ_tl_* threshold strain, together with a slight decrease in the γ_tv_ threshold strain, with increasing FF content was measured.
8. For the studied fRCA, the G_max_ values could also be determined from the bender element test, although only for the mixture with FF = 20% G_max, BE_ ≈ G_max, RC_. In the case of other FF contents, in one test, the results were higher for the resonant column testing, and in another, they were higher for the bender element testing.

## Figures and Tables

**Table 1:** A summary of the test results for a selection of the properties of recycled aggregates.

Description	Standard	Unit	Value
Sand index, SE	[26]	-	82
Shredding resistance, LA	[27]	%	39, LA_40_
Abrasion resistance, M_DE_	[28]	%	28, M_DE_35
Frost resistance, F	[29]	%	9.64, F10
CBR value	[30]	%	60
Flatness index, FI	[31]	%	42, FI_50_
Shape index, SI	[32]	%	23, SI_40_
Content of grains with crushed or broken surfaces	[33]	%	78
Grain density	[34]	Mg/m^3^	2.51
Water absorption	[34]	%	6.55
Bulk density (loose)	[27]	Mg/m^3^	1.41
Methylene blue	[35]	g/kg aggregate	1.66

**Table 2:** Main physical properties of the tested materials.

No	Mixture Code	Composition	G_S_ ^a^	d_50_ (mm)	C_u_ ^b^	Cc ^c^	e_min_ (-)	e_max_ (-)
1	M1	fRCA_0FF	2.61	0.20	2.91	0.91	0.863	1.091
2	M2	fRCA_10FF	2.61	0.16	2.86	1.27	0.719	1.077
3	M3	fRCA_20FF	2.61	0.14	5.15	1.29	0.784	0.955
4	M4	fRCA_30FF	2.61	0.12	5.60	1.17	0.699	0.882
5	M5	fRCA_5FF	2.61	0.21	2.36	1.01	0.677	0.951
6	M6	fRCA_15FF	2.61	0.18	4.88	2.49	0.716	1.023

^a^ Specific gravity. ^b^ Uniformity coefficient C_u_ = d_60_/d_10_. ^c^ Curvature coefficient C_c_ = d_30_^2^/(d_60_ × d_10_).

**Table 3:** Variation in small-strain shear modulus vs. variation in the fine fraction content of the tested mixtures.

	FF	p′ (kPa)		
		90	180	270
	air-dry mixtures			
ΔG_max_ (MPa)	0–10%	−3.99	3.19	3.97
	10–20%	25.88	22.72	23.49
	20–30%	23.31	50.96	60.37
	mixtures with OMC			
ΔG_max_ (MPa)	5–15%	−4.10	3.27	10.33

**Table 4:** Variation in the small-strain shear modulus vs. the variation in mean effective stress in the tested fRCA mixtures.

Lp.	Mixture	ΔG_max_ (MPa)	
	Air—Dry	Δσ _(180 kPa−90 kPa)_	Δσ _(270 kPa−180 kPa)_
1	M1	52.49	37.94
2	M2	45.31	37.16
3	M3	48.48	36.39
4	M4	20.83	26.97
	With OMC	Δσ _(180 kPa−90 kPa)_	Δσ _(270 kPa−180 kPa)_
5	M5	49.47	40.74
6	M6	42.10	33.67

**Table 5:** Variation in the small-strain damping ratio vs. the variation in mean effective stress in the tested fRCA mixtures.

	FF	p′ (kPa)		
		90	180	270
	air-dry mixtures			
ΔD_min_ (%)	0–10%	0.06	0.02	0.13
	10–20%	1.04	0.33	−0.09
	20–30%	1.47	1.74	1.63
	mixtures with OMC			
ΔD_min_ (%)	5–15%	0.07	0.08	−0.03

**Table 6:** Variation in small-strain damping ratio vs. the variation in mean effective stress in the tested fRCA mixtures.

Lp.	Mixture	ΔD_min_ (%)	
	Air—Dry	Δσ _(180 kPa−90 kPa)_	Δσ _(270 kPa−180 kPa)_
1	M1	0.13	0.00
2	M2	0.17	−0.11
3	M3	0.88	0.31
4	M4	0.61	0.42
	With OMC	Δσ _(180 kPa−90 kPa)_	Δσ _(270 kPa−180 kPa)_
5	M5	0.18	0.09
6	M6	0.20	−0.02

**Table 7:** Thresholds between the strain regimes for the tested fRCA mixtures.

Parameter	Linear Threshold Shear Strain (%)		Volumetric Cyclic Threshold Shear Strain (%)		
	γ_tl_^V^	γ_tl_^S^	γ_tv_^V^	γ_tv_^S^	γ_tv_^SC^
	Air-dry mixtures				
G/G_max_	6.00 × 10^−4^		1.00 × 10^−2^	2.00 × 10^−2^	2.80 × 10^−2^
D/D_min_		6.00 × 10−4		1.70 × 10^−2^	
	Mixtures with OMC				
G/G_max_	7.70 × 10^−4^		1.80 × 10^−2^	9.50 × 10^−3^	1.65 × 10^−2^
D/D_min_		3.80 × 10^−4^		1.50 × 10^−2^	

## Data Availability

The data presented in this study are available on request from the corresponding author, due to their size.
